# Update on Progress in Electronic Reporting of Laboratory Results to Public Health Agencies — United States, 2014

**Published:** 2015-04-03

**Authors:** Emilie Lamb, John Satre, Steve Pon, Glorietta Hurd-Kundeti, Bonnie Liscek, C. Jason Hall, Robert W. Pinner, Laura Conn, Julie Zajac, Megan Smallwood, Kaley Smith

**Affiliations:** 1 North Carolina Department of Health and Human Services; 2 Iowa Department of Health; 3 California Department of Public Health; 4 Kansas Department of Health & Environment; 5 Division of Preparedness and Emerging Infections; 6 National Center for Emerging and Zoonotic Infectious Diseases; 7 Office of Public Health Scientific Services; 8 Division of Health Informatics and Surveillance, Center for Surveillance, Epidemiology and Laboratory Services, CDC

Since 2010, CDC has provided resources from the Prevention and Public Health Fund of the Affordable Care Act (1) to 57 state, local, and territorial health departments through the Epidemiology and Laboratory Capacity for Infectious Diseases cooperative agreement to assist with implementation of electronic laboratory reporting (ELR)* from clinical and public health laboratories to public health agencies. To update information from a previous report (2) about the progress in implementing ELR in the United States, CDC examined regular communications between the agency and the 57 health departments during 2012–2014. The results indicated that, as of July 2014, 67% of the approximately 20 million laboratory reports received annually for notifiable conditions were received electronically, compared with 62% in July 2013. These electronic reports were received by 55 of the 57 jurisdictions and came from 3,269 (up from nearly 2,900 in July 2013) of approximately 10,600 reporting laboratories (Figure 1). The proportion of laboratory reports received electronically varied by jurisdiction (Figure 2). In 2014, compared with 2013, the number of jurisdictions receiving >75% of laboratory reports electronically was higher (21 versus 14), and the number of jurisdictions receiving <25% of reports electronically was lower (seven versus nine). National implementation of ELR continues to increase and appears it might reach 80% of total laboratory report volume by 2016.

Facilities of four large commercial laboratories† account for 39% of the total ELR volume, whereas public health laboratories account for 23%. Hospital laboratories, which number over 5,000, currently send 20% of ELR volume, an increase from 14% in 2013 (Figure 3).

As of July 2014, 479 hospital laboratories were using the message format§ required under the Centers for Medicare and Medicaid Services’ Meaningful Use incentive program to report clinical test results (3), compared with fewer than 200 in 2013. In addition, the number of hospital laboratories testing Meaningful Use–compliant ELR transmissions has more than doubled, to more than 1,300 as of July 2014. Nationally, nearly 3,000 eligible hospitals have registered their intent to send electronic laboratory reports to public health agencies under the Meaningful Use program.

Following are reports from four states that highlight some of their experiences with ELR.

## Iowa

ELR implementation has streamlined surveillance for reportable diseases at the Iowa Department of Public Health. For example, with ELR in place, the Iowa Department of Public Health handled a large outbreak of pertussis (1,738 cases) in 2012 and concurrent outbreaks of cryptosporidiosis (1,486 cases) and cyclosporiasis (148 cases) in 2013 without the need to divert additional staff members or resources from other public health activities. In contrast, during the 2006 national mumps outbreak (1,965 Iowa cases), before ELR was implemented in Iowa, the disease monitoring team required substantial temporary reassignment of staff members and temporary employees for data entry.

## North Carolina

In North Carolina, use of ELR has decreased the time required for case processing by as much as 5 days (from when a case report is received by public health authorities to when it is submitted to CDC). Additionally, cases initiated via ELR are more accurately reported and require less follow-up than cases initiated through traditional mechanisms, such as paper reporting of laboratory results. In 2013, 76% of all laboratory reports were received by the North Carolina Division of Public Health electronically compared with 56% in 2012, largely because of the integration of HIV and syphilis reporting via ELR into the North Carolina Electronic Disease Surveillance System.

## Kansas

In January 2012, the Kansas Department of Health and Environment implemented an integrated disease surveillance information system that supports ELR for all reportable diseases. As of October 2014, 29 laboratories were reporting electronically, resulting in 74% of all laboratory reports for notifiable conditions being received through ELR and arriving on average 2.7 days sooner than they did on paper faxes (a reduction from 6.0 days to 3.3 days).

## California

In October 2013, the California Department of Public Health implemented ELR within a secure, statewide integrated electronic disease reporting and surveillance system. The California Reportable Disease Information Exchange accepts ELR from a growing group of submitters, now including 305 clinical (hospital) laboratories, four health information exchanges, and eight electronic health record system vendors. The California Department of Public Health currently receives approximately 11,000 electronic reports weekly; over 90% of this volume is automatically processed into California Reportable Disease Information Exchange, eliminating the need for local health departments to input those laboratory reports manually.

### Discussion

National implementation of ELR continues to progress steadily, as evidenced by increases in both the number of laboratories using ELR and the proportion of reports being sent via ELR. Moreover, the examples from four states illustrate some of the impact ELR is having on public health practice.

The increases in the number of hospital laboratories using ELR and the proportion of reports sent via ELR by hospital laboratories suggest that the Meaningful Use program might be having an impact on national ELR implementation. The steep increase in the number of hospital laboratories testing ELR feeds bodes well for continued increases in the number of hospital laboratories transitioning to the use of ELR for public health reporting. However, moving new ELR feeds through the testing processes and into routine use can take several months. To help expedite this process, public health agencies can adopt more efficient processes for moving ELR feeds from testing to routine use, hospital laboratories can ensure the acceptability of ELR messages before engaging health departments, and laboratory information system vendors can include or improve ELR functionality in their systems.

Large laboratories continue to make up a substantial proportion of ELR volume, but a renewed focus on completing ELR implementation from these high-volume reporters could have a big impact. Two strategies that might be explored with large laboratories, and potentially others that report to multiple jurisdictions, are adoption of a single message that would be widely acceptable to public health jurisdictions and use of a hub as a single place to send to.

Adoption of a single message that would be widely acceptable to public health jurisdictions and use of a hub as a single place for large laboratories and potentially others who report to multiple jurisdictions are two strategies that might be explored.

ELR funding for public health agencies, coupled with CDC-provided ELR technical assistance appears to be resulting in increased implementation of ELR. The new CDC surveillance strategy also highlights ELR as a priority initiative for the agency (4). With sustained effort and funding, ELR implementation in the United States is on track to reach a target of 80% of laboratory reporting volume via ELR in 2016.

What is already known on this topic?

Electronic reporting of laboratory results to public health agencies can improve public health surveillance for reportable diseases and conditions.

What is added by this report?

As of July 2014, 67% of the approximately 20 million laboratory reports received annually for notifiable conditions in these jurisdictions were received electronically, compared with 62% in July 2013.

What are the implications for public health practice?

Progress in electronic laboratory reporting has resulted from a new emphasis and improved capacity and preparedness in health departments to address technical and policy issues. National implementation of ELR continues to progress steadily, as evidenced by increases in both the number of laboratories using ELR and the proportion of reports being sent via ELR.

## Figures and Tables

**FIGURE 1:**
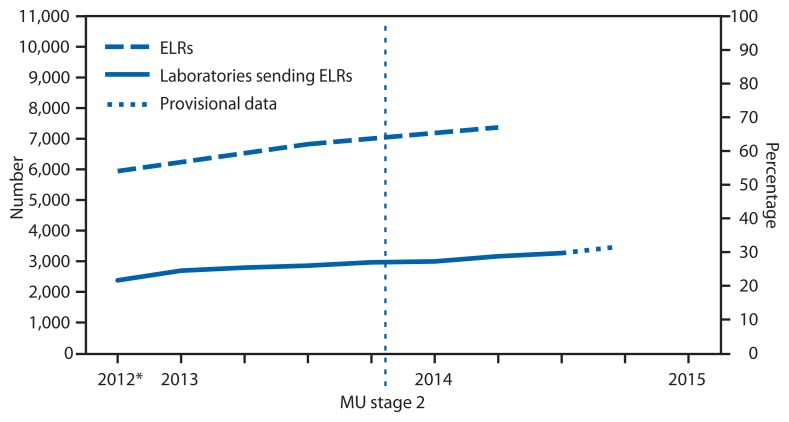
Number and percentage of laboratories sending electronic laboratory reports (ELRs) and number and percentage of reports that were sent electronically to public health agencies — United States, 2012–2014 **Abbreviation:** MU = Meaningful Use program of the Centers for Medicaid and Medicare Services. * As of the third quarter 2012.

**FIGURE 2:**
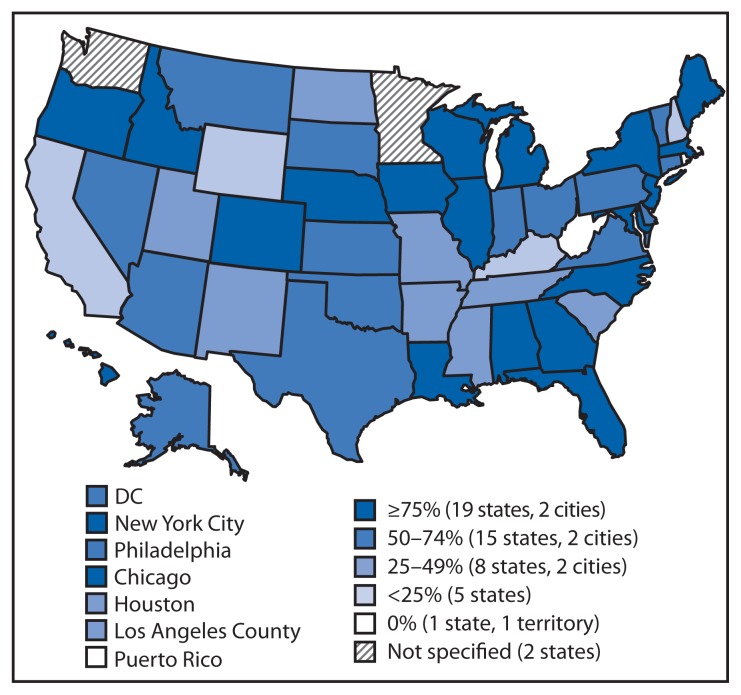
Percentage of U.S. laboratory reports received electronically, by public health jurisdiction — 57 jurisdictions, 2014

**FIGURE 3:**
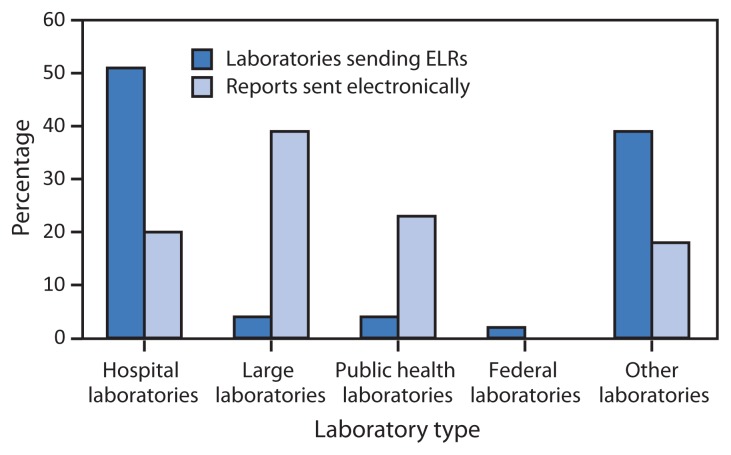
Percentage of laboratories sending electronic laboratory reports (ELRs) and percentage of reports sent electronically, by laboratory type — United States, April 2014

## Footnotes

* Electronic laboratory reporting (ELR) generally refers to the secure, automated messaging of laboratory reports, using HL7 or other formats, sent using one or more electronic communication protocols. Direct Web entry (the manual entering of reports over the Internet by laboratories but not through electronic messaging) is included in this report as ELR because it does not require manual data entry by public health agencies into a surveillance information system or into an ELR repository.

† LabCorp, Quest Diagnostics, ARUP Laboratories, and Mayo Clinic.

§ HL7 v2.5.1 Implementation Guide: Electronic Laboratory Reporting To Public Health (US Realm), Release 1 with Errata.
